# Implementation of automated reporting of estimated glomerular filtration rate among Veterans Affairs laboratories: a retrospective study

**DOI:** 10.1186/1472-6947-12-69

**Published:** 2012-07-12

**Authors:** Rasheeda K Hall, Virginia Wang, George L Jackson, Bradley G Hammill, Matthew L Maciejewski, Elizabeth M Yano, Laura P Svetkey, Uptal D Patel

**Affiliations:** 1 Center for Health Services Research in Primary Care, Veterans Affairs Health Services Research & Development, Durham, NC, USA; 2 Department of Medicine, Duke University School of Medicine, Durham, NC, USA; 3 Center for the Study of Healthcare Provider Behavior, Veterans Affairs Health Services Research & Development, Sepulveda, CA, USA; 4 Department of Health Services, University of California at Los Angeles, Los Angeles, CA, USA; 5 Sarah W. Stedman Nutrition and Metabolism Center, Duke University Medical Center, Durham, NC, USA; 6 Duke Clinical Research Institute, Duke University School of Medicine, Durham, NC, USA

## Abstract

**Background:**

Automated reporting of estimated glomerular filtration rate (eGFR) is a recent advance in laboratory information technology (IT) that generates a measure of kidney function with chemistry laboratory results to aid early detection of chronic kidney disease (CKD). Because accurate diagnosis of CKD is critical to optimal medical decision-making, several clinical practice guidelines have recommended the use of automated eGFR reporting. Since its introduction, automated eGFR reporting has not been uniformly implemented by U. S. laboratories despite the growing prevalence of CKD. CKD is highly prevalent within the Veterans Health Administration (VHA), and implementation of automated eGFR reporting within this integrated healthcare system has the potential to improve care. In July 2004, the VHA adopted automated eGFR reporting through a system-wide mandate for software implementation by individual VHA laboratories. This study examines the timing of software implementation by individual VHA laboratories and factors associated with implementation.

**Methods:**

We performed a retrospective observational study of laboratories in VHA facilities from July 2004 to September 2009. Using laboratory data, we identified the status of implementation of automated eGFR reporting for each facility and the time to actual implementation from the date the VHA adopted its policy for automated eGFR reporting. Using survey and administrative data, we assessed facility organizational characteristics associated with implementation of automated eGFR reporting via bivariate analyses.

**Results:**

Of 104 VHA laboratories, 88% implemented automated eGFR reporting in existing laboratory IT systems by the end of the study period. Time to initial implementation ranged from 0.2 to 4.0 years with a median of 1.8 years. All VHA facilities with on-site dialysis units implemented the eGFR software (52%, p<0.001). Other organizational characteristics were not statistically significant.

**Conclusions:**

The VHA did not have uniform implementation of automated eGFR reporting across its facilities. Facility-level organizational characteristics were not associated with implementation, and this suggests that decisions for implementation of this software are not related to facility-level quality improvement measures. Additional studies on implementation of laboratory IT, such as automated eGFR reporting, could identify factors that are related to more timely implementation and lead to better healthcare delivery.

## Background

Since its development, automated laboratory reporting of estimated glomerular filtration rate (eGFR) has facilitated diagnosis and management of chronic kidney disease (CKD) [1]. To report eGFR, clinical laboratories implement software that calculates eGFR from equations based on serum creatinine and demographic variables. As a result, clinicians who receive laboratory reports of eGFR in addition to serum creatinine may make more informed clinical decisions. The importance of automated eGFR reporting was established by National Kidney Foundation clinical guidelines in 2002, and, later, endorsed by the National Kidney Disease Education Program and International Society of Nephrology in subsequent years [2-4]. Despite these endorsements, automated eGFR reporting uptake has been slow; only 40% of U.S. clinical laboratories had eGFR reporting software by 2007 [5]. In other words, more than half of US laboratories had not implemented eGFR reporting by 2007, and physicians served by those laboratories may have missed opportunities for early detection of CKD. Because of the complications associated with late detection of CKD and increasing prevalence of CKD [6,7], it is important to understand the mechanisms associated with implementation of automated eGFR reporting. Implementation of automated eGFR reporting could be explained by organizational characteristics [8], but that relationship has not been studied to date.

For clinicians within the Veterans Health Administration (VHA), detection of CKD is important because the prevalence of CKD among veterans is expected to grow due to aging and increasing prevalence of diabetes and hypertension [6]. To enhance detection and management of CKD, the VHA adopted system-wide automated eGFR reporting by providing a software patch for existing laboratory information technology (IT) systems in 2004. The software patch was programming code that enabled each laboratory’s IT system to calculate eGFR with the Modification of Diet in Renal Disease (MDRD) equation and report eGFR values with every serum creatinine. To implement automated eGFR reporting, each laboratory had to install and activate the software patch. Although software patch implementation is typically issued as a mandate, for unclear reasons, implementation of this patch was left to the discretion of individual laboratories. Without a mandate or a central mechanism for simultaneous implementation in all laboratories, the VHA laboratories provide a natural experiment for observing implementation of automated eGFR reporting in a large, national, vertically-integrated healthcare system. Individual VHA laboratories may have rates of implementation of automated eGFR reporting that differ from non-VHA laboratories because the VHA is a highly integrated health system with a system-wide electronic health record and a strong reputation in quality performance [9]. This study examines the timing of implementation of automated eGFR reporting across the VHA. In addition, we sought to identify organizational characteristics that could explain the rate of implementation observed not only in the VHA, but also within laboratories in other healthcare systems.

## Methods

### Study Design and Population

We performed a retrospective observational study of VHA laboratories and their affiliated clinical facilities. The study population was derived from VHA community-based outpatient clinics (CBOCs) or Veteran Affairs medical centers (VAMCs) with available laboratory data between July 2004 and September 2009. VHA facilities that did not have complete laboratory and organizational data, or were not independent VHA facilities (e.g., skilled nursing facilities, rehabilitation centers, and domiciliaries) were excluded. This study was approved by the Institutional Review Board of the Durham Veterans Affairs Medical Center.

### Data Sources

Time to eGFR software implementation at each VHA laboratory was derived from administrative data in the VHA Decision Support System’s Laboratory Results National Data Extract (DSS-NDE) between July 2004 and September 2009. Each of the creatinine and eGFR laboratory results had an associated date on which the laboratory tests were measured. These dates were used to identify the presence and timing of eGFR implementation by VHA facility.

Organizational data come from several sources. The primary source of organizational data was the 2006 VHA Clinical Practice Organizational Survey-Chief of Staff Module (CPOS-COS survey), a survey of facility quality improvement (QI) characteristics and activities. Survey items were carefully selected to match organizational domains of our conceptual model (Table 1). Additional organizational characteristics were obtained from VHA administrative data. Facility complexity was captured through the 2005 VHA Facility Complexity Score Model, maintained by the VHA National Leadership Board. VHA facilities with dialysis services were identified through clinic-specific utilization codes in the DSS-NDE.

**Table 1:** Domains, Definitions, and Variables of the Conceptual Model of Implementation of eGFR reporting

**Domain**	**Definition**^**1**^	**Variable**
Facility context	Characteristics of the medical centers that may affect implementation	Number of acute care beds; Facility complexity score^2^; Affiliation with an academic medical center; Presence of nephrologists; Presence of a dialysis unit
Implementation activities and Structures	Approaches used to directly introduce, spread, and support the implementation	Use of clinical champions; Monitoring of guideline implementation; Fostering of collaboration among facilities; Presence of plan for implementation; Presence of teamwork for implementation; Available financial resources for implementation
Staff awareness and capabilities	Characteristics of staff responsible for implementing the innovation	Resistance from Primary Care Providers; Subspecialists; Local Support staff to QI

^1^ Definitions derived from Van Deusen Lukas’ conceptual model. Adapted from Table 2 of Reference [10]. ^2^ Facility complexity is a composite measure describing patient volume, level of intensive care services, patient risk, residency slots, research funds, and specialty physicians at VHA facilities.

### Measurement

We developed three eGFR implementation outcomes for each facility: 1) a dichotomous indicator of implementation status by September 2009; 2) time to initial implementation of automated eGFR reporting (between July 2004 and September 2009); and 3) time to full implementation of automated eGFR reporting (between July 2004 and September 2009). For time to initial implementation, time zero was defined as the date of VHA’s issued mandate for eGFR reporting to laboratory information managers (July 14, 2004). Based on administrative data, some facilities appeared to intermittently report eGFR values after time zero before consistently reporting eGFR per the mandate. Thus, initial implementation date was based on consistent eGFR reporting, with consistency attained when at least 1% of the median creatinine tests per month were accompanied by a reported eGFR. Time to full implementation was defined as the time between initial implementation and the day when at least 90% of median creatinine tests per month were accompanied by a reported eGFR. Lastly, facilities were categorized into implementation stages (early, mid, and late-stage) based on tertile distributions of time to initial implementation. Facilities that did not implement eGFR reporting by the end of our observation period were grouped separately.

To identify organizational characteristics associated with implementation of automated eGFR reporting, we selected a conceptual model that has been used in prior studies to evaluate organizational characteristics associated with implementing new clinical innovations within the VHA. We applied Van Deusen Lukas and colleagues’ conceptual model of implementation, which posits that implementation of a clinical innovation is influenced by 1) *facility context*, 2) *implementation activities and structures*, and 3) *staff awareness and capabilities*[10]. Then, we identified variables to match this conceptual model to test for association with implementation of automated eGFR reporting (Table 1). The *facility context* domain includes characteristics of each VHA facility, such as availability and demand for specific clinical services, which may affect implementation. The next domain, *implementation activities and structures*, assesses the use of methods that facilitate spread of implementation. Variables for this domain were CPOS-COS survey questions that measured methods for QI and clinical practice guideline implementation (Table 2). Each of the methods selected have been considered important facilitators of QI activities [11]. Other CPOS-COS survey QI activities were not included in our primary analysis because they did not seem relevant to implementation of automated eGFR reporting. Instead, these QI activities were included in a sum to assess for an association between total QI activities and implementation of automated eGFR reporting. The final domain of the conceptual model, *staff awareness and capabilities*, acknowledges that staff opinions on the implementation of automated eGFR reporting are important. Variables for this domain are derived from CPOS-COS survey questions (Table 2).

**Table 2:** Survey items extracted from the VHA clinical practice organizational survey chief of staff module

**Survey Item**	**Response Options**
How many active acute care beds are in each major bedsection of your hospital?	Numeric response
Does your VA have an academic medical school training program for residents?	Yes/No
Which of the following types of specialty-trained physicians (Nephrologists) do you have onsite at your VA?	Yes/No
To what extent do you think each of the following serves as a barrier to improving performance at your facility?	Not a barrier; Small barrier; Moderate barrier; Large Barrier
Resistance from Primary Care providers	
- Resistance from Subspeciality providers	
- Resistance from Local Support staff	
- Limited financial resources to support needed changes	
To what extent has your facility implemented the following actions to improve your VA’s clinical performance?	Not at all; Very little; Some; Great; Very great
Designated a site champion for specific clinical guidelines or performance measures	
- Monitored the pace at which guidelines were implemented across the facility	
Fostered collaboration among facilities in guideline implementation within the facility	
In the past year, when clinical practice guidelines were implemented in your facility, to what extent:	Not at all; Very little; Some; Great; Very great
Did teamwork exist at your facility in implemented the guidelines?	
Were key implementation steps planned?	

CPOS-COS survey questions were deliberately selected to match the domain definitions of the conceptual model. Although these questions are not specific to automated eGFR reporting, they have been validated for general QI innovations (unpublished data from co-author Elizabeth M. Yano). Data for each variable was obtained from Likert scale questions by collapsing four-level or five-level survey responses into dichotomous variables. The distribution of responses to the five-level survey questions was examined to classify the middle level responses into either dichotomous variable. As dichotomous variables, the survey responses were interpreted as the presence or absence of the organizational characteristics of interest.

### Analysis

To detect variation in time to initial implementation and time to full implementation of eGFR reporting, we examined univariate distributions. To identify differences by implementation status and between implementation stages, we conducted ANOVA and t-tests for continuous variables and Chi-square or Fisher’s exact tests for categorical variables. We performed simple logistic regressions to determine the strength of association between each organizational characteristic and implementation of eGFR reporting. To test for association with time to implementation, survival analysis was performed using proportional hazards models. Variables forced into the multivariable regression model included presence of a dialysis unit, presence of nephrologists, and affiliation with an academic medical center. A backwards elimination procedure was performed to fit additional variables into the model if the significance level was less than 0.2. Nonparametric tests were applied when data were not normally distributed. Alpha levels for statistical significance were set at 0.05.

## Results

### Implementation Rates

**Figure 1:**
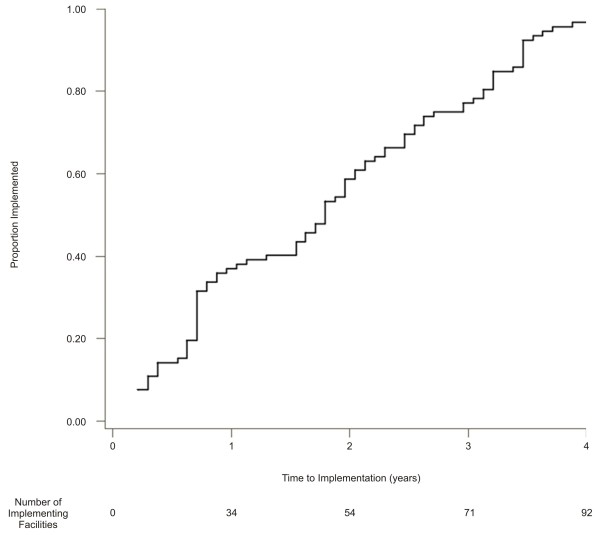
**Proportion of Facilities that Implemented Automated eGFR Reporting over time.** For the facilities that implemented automated eGFR reporting during the study period (n=92), this line plot depicts time to initial implementation measured in years from the date of software availability.

Although we identified 135 eligible facilities, only 104 facilities had complete organizational data, so 31 facilities were excluded from the analyses. Of the 104 facilities included, 92 (88%) facilities implemented eGFR reporting by the end of the study period. Time to initial implementation varied widely, from 0.2 to 4.0 years (median=1.8 years) from the VHA’s initial mandate for adoption (Figure 1). Among the excluded facilities, 18 of 31 (58%) implemented automated eGFR reporting with a similar median time to initial implementation (1.6 years), while the remaining facilities did not implement.

After the initial implementation of eGFR reporting software, several months passed before eGFR values were automatically reported consistently with all creatinine results at many facilities. The median time between initial implementation and full implementation (defined as the time between initial implementation and the date when ≥90% of median creatinine tests were accompanied by a reported eGFR) was 0.8 years (IQR=0.42). By the end of the study period, 5 of the 92 facilities that initiated the software did not attain full implementation.

### **Organizational characteristics associated with implementation**

*Facility context* was associated with implementation of automated eGFR reporting to some extent. Facilities operating a dialysis unit were more likely to implement automated eGFR reporting (52%, p<.001) (Table 3). However, implementation status was not associated with facility size (e.g., number of beds), facility complexity, presence of practicing nephrologists, or academic affiliation.

**Table 3:** Organizational characteristics by implementation status

**Characteristic**	**Overall N=104**	**Implemented N=92**	**Not Implemented N=12**	**P Value**	**Unadjusted Odds of Implementation**^1^
*Facility context*					
Number of Acute Care Beds (mean±SD)	115.9±87.4	118.5±89.4	96.7±70.1	0.42	
Facility Complexity Score^2^					
Level 1	56 (54)	50 (89)	6 (11)	0.86	1.19 (0.36-3.97)
Level 2	20 (19)	17 (85)	3 (15)		0.68 (0.16-2.78)
Level 3	28 (27)	25 (89)	3 (11)		1.12 (0.28-4.47)
Presence of Nephrologist	72 (69)	64 (89)	8 (11)	1.00	1.14 (0.32-4.11)
Presence of Dialysis Unit	54 (52)	54 (100)	0 (0)	<0.001	35.39 (2.03-615.93)
Affiliation with Academic Medical Center	84 (81)	72 (86)	8 (14)	0.12	0.14 (0.01-2.49)
*Implementation activities and structures*					
Use of Clinical Champions^3^	93 (90)	81 (87)	12 (13)	0.60	0.31 (0.02-5.64)
Monitoring of Guideline Implementation	89 (86)	78 (89)	11 (12)	0.52	0.51 (0.06-4.24)
Presence of Plan for Implementation	98 (94)	86 (88)	12 (12)	1.00	0.53 (0.03-10.04)
Fostering of Collaboration among facilities^3^	91 (88)	80 (88)	11 (12)	0.70	0.66 (0.08-5.63)
Presence of Teamwork for Implementation	100 (96)	88 (88)	12 (12)	1.00	0.79 (0.04-15.51)
Adequate Financial Resources	30 (29)	25 (83)	5 (17)	0.30	0.52 (0.15-1.80)
*Staff awareness and capabilities*					
Resistance from Primary Care Providers	17 (16)	17 (100)	0 (0)	0.20	5.79 (0.33-102.62)
Resistance from Subspecialists	20 (19)	20 (100)	0 (0)	0.11	7.07 (0.40-124.54)
Resistance from Local Support Staff	8 (8)	8 (100)	0 (0)	0.59	2.51 (0.14-46.32)

Values represented are frequency (percentage). ^1^Odds ratio (95% confidence interval).

^2^ Complexity level 1 facilities had highest complexity and level 3 the lowest.^3^ N=103 for this survey question.

Measures of *implementation activities and structures* were favorable for most VHA facilities. A majority of facilities (>85%) utilized clinical champions and monitoring of guideline implementation (general measure across conditions, not specific to CKD treatment), and there were no statistically significant differences by implementation status (p=0.60 and p=0.52, respectively) (Table 3). Similarly, the use of key implementation steps and collaboration between facilities were common for both facilities that implemented the software and facilities that did not implement (p=1.00 and p=0.70, respectively). Only a minority of facility chiefs of staff (29%) perceived adequacy of financial resources to support innovations, and this perception was similar in both groups (p=0.30). Regardless of implementation status, there was high use of QI activities, median=10 (maximum=11, IQR=3 for facilities that did implement, IQR=1.5 for facilities that did not implement, p=0.27). For measures of *staff awareness and capabilities*, resistance from primary care, subspecialists, and support staff as a barrier to QI was uncommon, and these measures also did not differ by implementation status (p=0.20, p=0.12, and p=0.59, respectively). Odds ratios (shown in Table 3) revealed no significant associations between the variables derived from the CPOS-COS survey and implementation of automated eGFR reporting.

To relate timing of implementation to organizational characteristics, facilities were categorized into stages of implementation, early, mid, or late stage with median time to implementation of 0.6, 1.8, and 3.2 years, respectively. In our examination of these stages, early stage facilities tended to be associated with more acute care beds, higher facility complexity scores, and more nephrology services compared to mid stage and late stage facilities (Table 4). However, none of these measures of *facility context* were significantly different between these groups or in a comparison of early and late stage facilities. In addition, there were no significant differences between facility stages for measures of *implementation activities and structures* (including total QI activities) and *staff awareness and capabilities*.

**Table 4:** Organizational characteristics by stage of initial implementation

**Characteristics**	**Stage N=31**	**Mid Stage N=30**	**Late Stage N=31**	**P Value**
*Facility Context*				
Number of Acute Care Beds^1^	123.6±91.2	117.1±93.0	114.9±86.9	0.93
Facility Complexity Score^2^				
Level 1	19 (61)	18 (60)	13 (42)	0.51
Level 2	5 (16)	4 (13)	8 (26)	
Level 3	7 (23)	8 (27)	10 (32)	
Presence of Nephrologists	23 (74)	22 (73)	19 (61)	0.47
Presence of Dialysis Unit	21 (68)	18 (60)	15 (48)	0.30
Affiliation with Academic Medical Center	25 (81)	25 (83)	22 (71)	0.46
*Implementation activities and structures*				
Use of Clinical Champions^3^	27 (90)	27 (90)	27 (87)	0.92
Monitoring of Guideline Implementation	27 (87)	26 (87)	25 (81)	0.73
Presence of Plan for Implementation	29 (94)	29 (97)	28 (90)	0.87
Fostering of Collaboration among facilities^3^	27 (87)	28 (93)	25 (83)	0.49
Presence of Teamwork for Implementation	29 (94)	29 (97)	30 (97)	1.00
Adequate Financial Resources	6 (19)	11 (37)	8 (26)	0.31
*Staff awareness and capabilities*				
Resistance from Primary Care Providers	7 (23)	3 (10)	7 (23)	0.35
Resistance from Subspecialists	7 (23)	4 (13)	9 (29)	0.33
Resistance from Local Support Staff	4 (13)	1 (3)	2 (7)	0.40

^1^ Mean (Standard deviation); all other values represented are frequency (percentage). ^2^ Complexity level 1 facilities had highest complexity and level 3 the lowest.^3^ N=103 for this survey question.

Unadjusted hazard ratios of the organizational characteristics revealed no associations with time to implementation of automated eGFR reporting. In a multivariable model for time to implementation, “presence of teamwork for implementation” was retained in a model with three other variables (presence of dialysis unit, presence of nephrologist, and affiliation with academic medical center), although this organizational characteristic was not statistically significant in the full model.

## Discussion

Despite an emphasis on quality performance and a system-wide electronic health record, implementation of automated eGFR reporting among VHA laboratories was incomplete and varied over many years following its adoption mandate. This wide variation could not be explained by facility-level organizational characteristics as there were no significant differences in these characteristics between the stages of implementation. There was only one significant difference by implementation status: presence of dialysis services was associated with implementation. Importantly, delayed or absent implementation of automated eGFR reporting could have translated into missed opportunities for earlier diagnoses of CKD among US veterans. Also, these findings illustrate that implementation of laboratory IT is not associated with level of healthcare system integration or presence of facility-level QI characteristics.

Rates of implementation of automated eGFR reporting have been evaluated in other studies. A survey from non-VHA settings in 2007 revealed that 40% of U.S. clinical laboratories reported eGFR with creatinine values in 2007 [5].That same year, the College of American Pathologists’ Annual Survey revealed eGFR reporting was used in approximately 50% of laboratories [12]. In the current study, we found that approximately 68% of VHA laboratories were reporting eGFR by 2007. Although these approximations of automated eGFR reporting usage were obtained by different methods, they all show a similar trend of incomplete implementation many years from initial software availability and consensus recommendations. This data suggests VHA laboratories had higher prevalence of automated eGFR reporting than non-VHA laboratories in 2007, and this may be explained by the VHA’s highly integrated healthcare system and electronic health record. Although when compared to another large integrated healthcare system, Alberta Health Services in Alberta, Canada, VHA laboratories had gradual implementation. In 2004, both healthcare systems adopted eGFR reporting software, but only Alberta Health Services had complete implementation of automated eGFR reporting software in its laboratories within that year [13]. Given that implementation of this innovation can vary regardless of system integration, more specific organizational characteristics likely explain this variation.

We sought to identify specific organizational characteristics that describe the variation in implementation of automated eGFR reporting within the VHA. Our analyses revealed that facilities that implemented the software were more likely to provide dialysis services than facilities that did not implement. In the VHA, facilities with dialysis units typically offer tertiary care services, and the demands of a more complex patient population and the better availability of financial resources in these facilities differs from facilities that provide only primary care services. As a result, the association of dialysis services to implementation of automated eGFR reporting suggests that presence of dialysis services may be highly correlated with one or all of the following: 1) presence of other resources in a facility, such as experienced laboratory information technology staff; 2) high demand to provide complex medical services (so facility is more apt to incorporate new innovations); or 3) efficient chain of command (facilities with dialysis units are larger and may incorporate more organizational hierarchy that promotes better accountability to tasks). Overall, the association of implementation of automated eGFR reporting and presence of dialysis services is consistent with previous research that indicates environmental barriers, such as lack of resources, may reduce provider adherence to clinical practice guidelines [14].

Aside from this finding, none of the other organizational characteristics were associated with implementation of automated eGFR reporting. These null results may have occurred because the variables used to describe the organizational domains of the conceptual model were only surrogate measures of implementation of automated eGFR reporting (Table 1). For example, the use of clinical champions is an important tool for implementation of healthcare innovations [15], and the CPOS-COS survey item for this variable was examined as a surrogate measure to determine whether use of clinical champions is important for implementation of automated eGFR reporting. All of the facilities reported similar frequencies in use of clinical champions for general QI initiatives. Although these similarities exist for general QI initiatives, the survey didn’t solicit the opinions of laboratory personnel to directly assess the use of clinical champions in implementation of automated eGFR reporting. As a result, the importance of clinical champions cannot be ruled out because our methods don’t include direct measurement of this variable. Similarly, all other organizational variables and domains from the conceptual model should be measured more directly before one concludes that there is no association with implementation of automated eGFR reporting. A similar survey distributed to laboratory personnel could provide more direct and specific assessment of these organizational variables. Not only that, direct query of laboratory decision-making processes or laboratory and IT leadership opinions, could provide more insight beyond the domains of this conceptual model.

We did not expect to find that none of the facility level characteristics from the CPOS-COS survey were associated with time to implementation of automated eGFR reporting. Intrinsic to our study design, we used the CPOS-COS survey for facility-level organizational variables because other similar studies demonstrated variable performance and practice patterns among VHA facilities that are related to facility- and clinic-level organizational characteristics [10,16-21]. We further justified use of the CPOS-COS survey because general QI characteristics can affect implementation of new innovations in individual facilities [22]. Conversely, our results show that general QI characteristics were not predictive of implementation of automated eGFR reporting. Automated eGFR reporting and other laboratory IT innovations are implemented differently from clinical innovations and are less affected by the extent of clinical QI infrastructure. This is an important lesson for future studies that attempt to assess the role facility-level organizational characteristics have in implementation of laboratory IT innovations.

This study has limitations that should be acknowledged. First, we did not assess laboratory-specific organizational characteristics for association with implementation of automated eGFR reporting. Without laboratory-specific variables, an explanation for the delayed implementation of automated eGFR reporting remains unclear. Second, we could not use verified start dates (of implementation) for each facility because of data transmission errors within the administrative data. As a result, we derived a definition for initiation of automated eGFR reporting that provided approximate dates of implementation. Last, the retrospective study design limited the data available for analysis of the process of software implementation. To counter this limitation, we utilized the 2006 CPOS-COS survey responses to obtain organizational characteristics that were measured within two years of the VHA’s initial adoption of automated eGFR reporting in 2004.

The wide variation in implementation of automated eGFR reporting in the VHA and other laboratories draws attention to the ongoing need for quality improvement in CKD management. Over the past decade, there has been interest in improving CKD identification, and automated eGFR reporting has been endorsed as a tool to enhance detection of CKD [2-4]. In fact, many studies, as described in a systematic review, have shown that automated eGFR reporting is associated with earlier detection of CKD [1]. Consequently, CKD detection may have been delayed in some VHA facilities as a result of the wide variation in implementation of automated eGFR reporting. This potential delay in diagnoses could be associated with disparate health outcomes between veterans who receive care at eGFR reporting facilities and those who do not. Specifically, late diagnosis of CKD leads to late nephrology referral which has been associated with increased mortality among those who progress to kidney failure [23]. Because of inconsistent implementation of automated eGFR reporting in other U.S. laboratories, disparate health outcomes may also exist outside the VHA. Given the growing prevalence of CKD, a concerted effort to enhance early detection and management of CKD remains important to prevent adverse outcomes and slow disease progression [6,24].

This study also has implications for future laboratory reporting innovations in nephrology. Although laboratories currently report eGFR from the MDRD equation, the MDRD equation may eventually be replaced by newer estimating equations, such as the Chronic Kidney Disease Epidemiology Collaboration (CKD-EPI) equation or estimates based on alternative biomarkers (e.g., cystatin-C) [25,26]. Alternative equations for estimation of GFR will necessitate the development and activation of additional software patches in clinical laboratories in the VHA and worldwide. Without a clear perspective on predictors of implementation, widespread implementation of additional eGFR reporting equations may be delayed.

Although we did not identify organizational characteristics clearly associated with rate of implementation of automated eGFR reporting, further investigation is warranted to inform implementation of future laboratory IT innovations which may lead to timely implementation and improved disease management. Future studies could include a qualitative analysis of facilities that did not implement automated eGFR reporting to reveal barriers to implementation. These barriers could be further evaluated prospectively with the implementation of similar laboratory IT. Additionally, an effort to develop a conceptual model specific for laboratory IT could enhance further implementation research.

## Conclusions

There is evidence that VHA laboratories, in addition to non-VHA laboratories, demonstrated a variable rate of software implementation for automated eGFR reporting. The rationale for this remains unclear, and it may be explained by future studies that evaluate laboratory-specific organizational characteristics. Without better awareness of barriers to implementation of automated eGFR reporting, a similar pattern of implementation may occur with future laboratory IT innovations which may limit their effectiveness in medical decision-making.

## Competing interests

The authors declare that they have no competing interests.

## Authors' contributions

RH was responsible for study design, data collection, statistical analysis, interpretation of results, and manuscript preparation. VW participated in study design, interpretation of results, and revision of the manuscript. GJ participated in study design, interpretation of results, data acquisition, and revision of the manuscript. BH assisted with interpretation of results. MLM participated in study design and interpretation of results. EMY participated in study design, data acquisition, and interpretation of results. LS participated in study design and revision of the manuscript. UDP participated in study design, interpretation of results, and revision of the manuscript. All authors read and approved the final manuscript.

## Disclaimer

The views expressed in this article are those of the authors and do not necessarily reflect the position or policy of the Department of Veterans Affairs or the United States government.

## Pre-publication history

The pre-publication history for this paper can be accessed here:

http://www.biomedcentral.com/1472-6947/12/69/prepub
